# Hydrocoel morphogenesis forming the pentaradial body plan in a sea cucumber, *Apostichopus japonicus*

**DOI:** 10.1038/s41598-022-09691-y

**Published:** 2022-04-11

**Authors:** Sumio Udagawa, Takafumi Ikeda, Kohei Oguchi, Hisanori Kohtsuka, Toru Miura

**Affiliations:** 1 Misaki Marine Biological Station, School of Science, The University of Tokyo, Misaki, Miura, Kanagawa 238-0225 Japan; 2 Department of Biological Sciences, Graduate School of Science, The University of Tokyo, Bunkyo, Tokyo 113-0033 Japan; 3 National Institute of Advanced Industrial Science and Technology (AIST), Tsukuba, Ibaraki 305-8566 Japan

**Keywords:** Developmental biology, Evolution, Evolutionary developmental biology

## Abstract

Echinoderms constitute an animal phylum characterized by the pentaradial body plan. During the development from bilateral larvae to pentaradial adults, the formation of the multiple of five hydrocoel lobes, i.e., the buddings from the mesodermal coelom, is the firstly emerging pentameral character. The developmental mechanism underlying the hydrocoel-lobe formation should be revealed to understand the evolutionary process of this unique and highly derived body plan of echinoderms, although the morphogenetic mechanisms of hydrocoel lobes are largely uninvestigated. In this study, using the sea cucumber *Apostichopus japonicus,* in which hydrocoel is easily observable, the developmental process of hydrocoel lobes was described in detail, focusing on cell proliferation and rearrangement. Cell proliferation was not specifically distributed in the growing tips of the hydrocoel lobes, and inhibition of cell proliferation did not affect lobe formation. During lobe formation, the epithelium of the hydrocoel lobes was firstly thickened and then transformed into a simple epithelium, suggesting that tissue expansion via tissue remodeling contributes to the hydrocoel-lobe formation.

## Introduction

Species belonging to the phylum Echinodermata constitute a deuterostome clade that shows unique pentaradial body plans. Echinoderms are thought to have evolved from a bilateral ancestor^1^. Therefore, the developmental process forming the pentaradial symmetry is an important issue to understand the diversification of animal forms^2^, although the developmental mechanisms underlying the establishment of the pentaradial body plan remain to be elucidated^3^.

In both extant and fossil species, the primary body axis of echinoderms is defined based on the arrangement of the ambulacrum, a region associated with the water vascular system^4^. The water vascular system is a synapomorphy of echinoderms that is involved in locomotion, circulation and feeding. The water vascular system exhibits a pentaradial structure, which forms a tubular network consisting of a ring canal around the mouth and radial canals that extend from the ring canal^5^. Thus, the water vascular system is an essential key character for considering the echinoderm body plans^6^.

**Figure 1:**
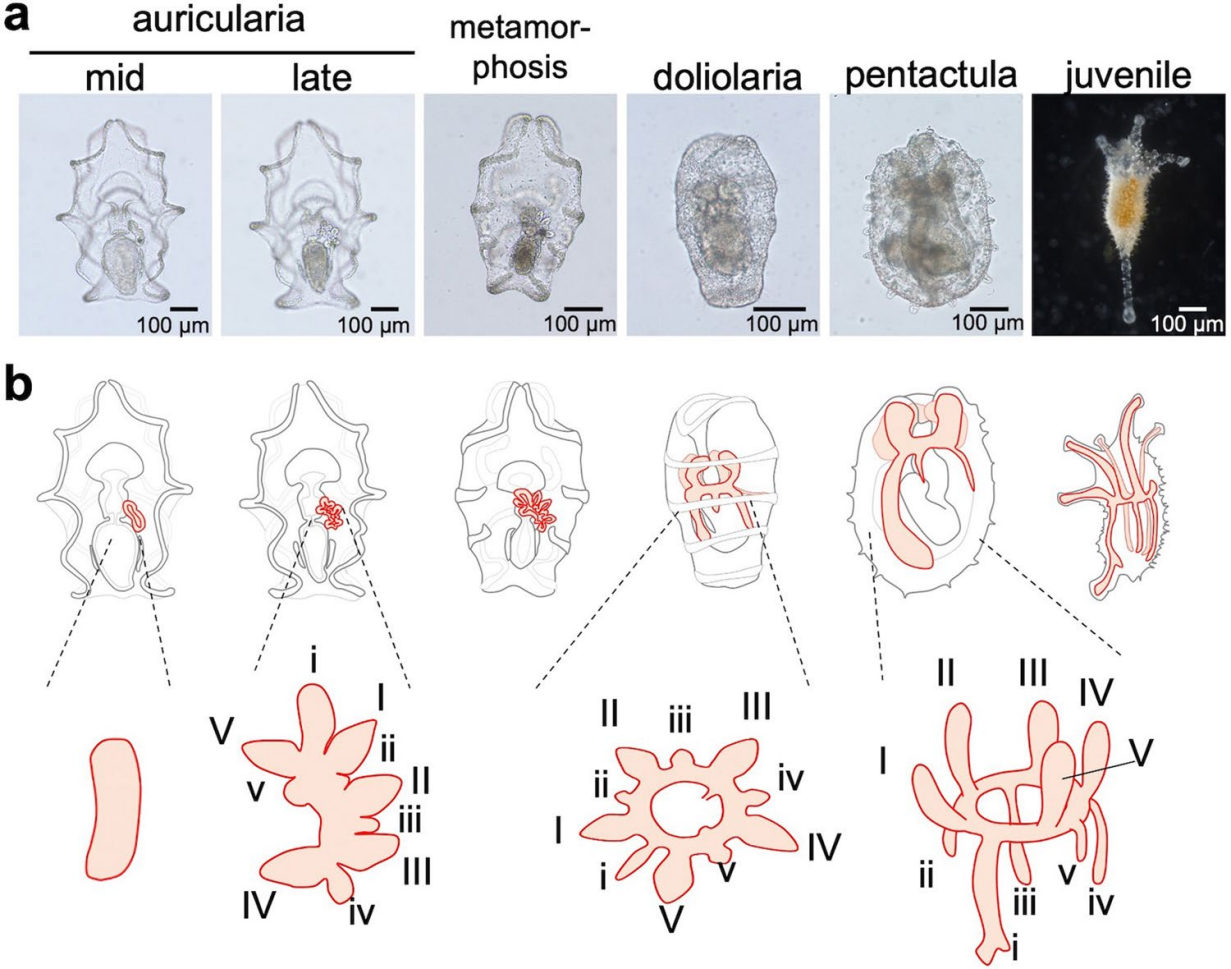
Normal development of the sea cucumber *Apostichopus japonicus* and schematic illustration of its hydrocoel. (**a**) The developmental process during auricularia larva and juvenile stages. Development proceeds from left to right. Auricularia, doliolaria, metamorphosis and pentactula are ventral views, anterior to the top, and juvenile is dorsal view, anterior to the top. (**b**) Schematic illustration of hydrocoel in each developmental stage. The orange part represents the hydrocoel or its derivative, i.e., the water vascular system. Auricularia and metamorphosing larvae are ventral views, and doliolaria, pentactula and juveniles are left lateral views, anterior to the top. The hydrocoel emerges on the left side of the archenteron in an auricularia, and 10 lobes are formed from the hydrocoel. After the lobe formation, the hydrocoel becomes horseshoe-like shaped to form a ring canal surrounding the digestive tract. Then, the larva metamorphoses into a doliolaria. Five of the lobes (I–V) extend anteriorly and become the water vascular canals of the tentacle of a juvenile, and another five lobes (i–v) are formed between the water vascular canals of the tentacle extend posteriorly and become radial canals. The numbering identities for hydrocoel lobes are based on the application of Loven’s axis to sea cucumbers^14^.

The water vascular system is formed from the hydrocoel, i.e., a mesodermal coelom that emerges on the left side of bilateral larvae^5,7^. In echinoderms, bilateral larvae are transformed into juveniles with pentaradial body plans through metamorphosis. At the time of metamorphosis, multiples of five buds (hydrocoel lobes) are projected from the hydrocoel, and the whole hydrocoel encircles the mouth, forming a ring and resulting in a pentaradial water vascular system (Fig. 1). It is known that in many echinoderm species, the emergence of multiples of five lobes on the hydrocoel is the first pentameral character in the ontogeny. The outline of hydrocoel development has so far been described in many echinoderms, and recent advances in microscopy have enabled us to observe the detailed structures inside an embryo^8^. However, the morphogenetic mechanisms underlying hydrocoel formation, especially histological transitions and behaviors of hydrocoel cells, have not yet been unraveled.

Although many of the studies on echinoderm development have been carried out in sea urchins ^9^, no studies have so far been conducted on the morphogenetic processes of hydrocoel-lobe formation in sea urchins due to their biological characteristics. For example, in *Strongylocentrotus purpuratus* showing indirect development, it takes a month from fertilization to the hydrocoel-lobe formation and its hydrocoel overlaps with the vestibule invagination which contributes to the adult rudiment^10^, making observations on the hydrocoel difficult. On the other hand, sea cucumbers (Holothuroidea), such as *Apostichopus japonicus*, showing indirect development, are suitable models to observe hydrocoel development since their larvae are transparent and do not form hydrocoel-associated structures such as adult rudiment, so the hydrocoel-lobe formation is easily observable.

The hydrocoel of *A. japonicus* is formed from the archenteron on the left side of the auricularia larva at approximately 4 days postfertilization (dpf). The larvae develop relatively quickly as indirect developers, so the hydrocoel lobes are formed on the hydrocoel at approximately 10 dpf^11,12^. In this species, as in other holothurians, 10 hydrocoel lobes are formed on the hydrocoel. Five major lobes (I-V) develop into water vascular canals of tentacles. Another five minor lobes (i-v), which are formed between each of the major lobes, extend posteriorly and develop into radial canals of the water vascular system (Fig. 1b)^13,14^. The hydrocoel becomes horseshoe-like shaped after the lobe formation and then forms the ring canal surrounding the digestive tract to establish the pentaradial structure. At the same time of ring canal formation, metamorphosis starts and the whole larval body is rearranged to become a doliolaria larva.

After the hydrocoel-lobe formation, pentaradial structures of adult sea cucumber, such as longitudinal muscle bands and radial nerve cords, are formed along the radial canals, which are formed from hydrocoel lobes^15–17^. Other echinoderms, such as sea urchins, also form pentaradial adult tissues around the hydrocoel^5^, which suggests that the hydrocoel lobes might induce the formation of pentaradial structures of echinoderms and contribute to the establishment of the pentaradial body axis also in these species. Thus, the spatial control of hydrocoel-lobe formation and the determination of the number of hydrocoel lobes is thought to be critical to the development of the pentaradial body plan.

In general, histological changes during morphogenesis involve changes in cell number, cell shape and cell arrangement^18^. In particular, branching or budding morphogenesis is driven by the contribution of cell proliferation and collective migration to the morphogenesis^19^. Therefore, it was considered that either, or both, of these cellular processes should be involved in the hydrocoel-lobe formation.

In this study, therefore, to reveal the morphogenetic process underlying the hydrocoel-lobe formation in *A. japonicus*, detailed observations at the cellular level were carried out focusing on changes in cell proliferation and cell arrangement during the formation process.

## Results

### Staging of hydrocoel-lobe formation

**Figure 2:**
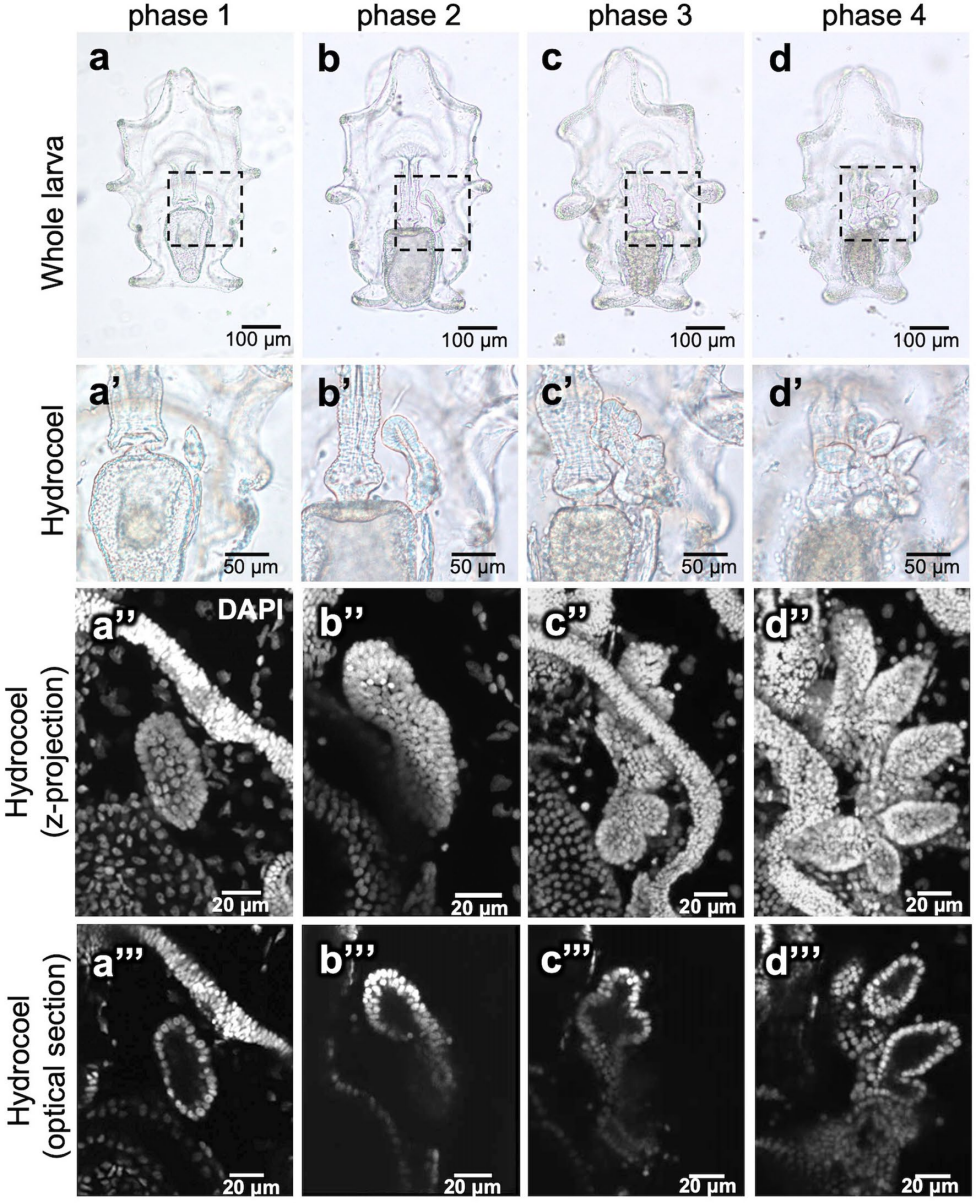
Overall morphology of the developing hydrocoel lobes. (**a–d**) Whole embryos under the microscope (ventral view, anterior to the top) from phase 1 to phase 4. (**a’–d’**) show the hydrocoel of a-d. The dotted lines in (**a–d**) indicate the region shown in (**a’–d’**). The hydrocoel of the specimens stained with DAPI is shown in (**a’’–d’**: z-stack images) and (**a’’’–d’’’**: optical sections). In phase 1, the hydrocoel was spindle shaped and then extended up to 100 µm along the longitudinal axis in phase 2. The hydrocoel lobes started to form in phase 3, and the lobes extended in phase 4. During phases 1 and 4, no significant change in the overall morphology of entire larvae other than the change in the hydrocoel was observed.

First, the overall process of hydrocoel-lobe formation in auricularia larvae was observed, focusing on morphological characteristics. Fixed specimens were observed under an optical microscope and a confocal laser scanning microscope after staining with 4',6-diamidino-2-phenylindole (DAPI) (Fig. 2). During the formation process, four developmental phases were defined based on the hydrocoel morphology (Table 1).

**Table 1:** Features of hydrocoel tissue in each phase of the hydrocoel-lobe morphogenesis.

Phase	Feature
Phase 1	Spindle shape (hydrocoel lobes are not formed) Single layered cell sheet
Phase 2	Bean shape (hydrocoel lobes are not formed) Multilayered cell sheet
Phase 3	Emergence of the hydrocoel lobe Single layered at the tip of the hydrocoel lobes
Phase 4	Extension of the hydrocoel lobe Single layered in the entire hydrocoel lobes

- *Phase 1* The hydrocoel primarily emerged on the left side of the digestive tract, showing a spindle-like shape (Fig. 2a–a’’’).
- *Phase 2* The entire hydrocoel extended up to 100 µm along its longitudinal axis of the spindle shape and became a slightly curved columnar shape (Fig. 2b–b’’’).
- *Phase 3* The hydrocoel lobes started to form (Fig. 2c–c’’’). The lobes were formed in a line along the AP axis of a larva. Each of the five minor hydrocoel lobes that became radial water vascular canals were respectively formed between the five major hydrocoel lobes that became water vascular canals of tentacles.
- *Phase 4* The lobes continuously extended to form tubular structures (Fig. 2d–d’’’). Each hydrocoel formed a tubular structure surrounded by epithelial tissue, and these tubular structures later formed the tubular network of the water vascular system.

There were no other obvious morphological changes in other parts of an auricularia larva, such as the digestive tract and ciliate bands, throughout these phases.

### Observations of cell proliferation

**Figure 3:**
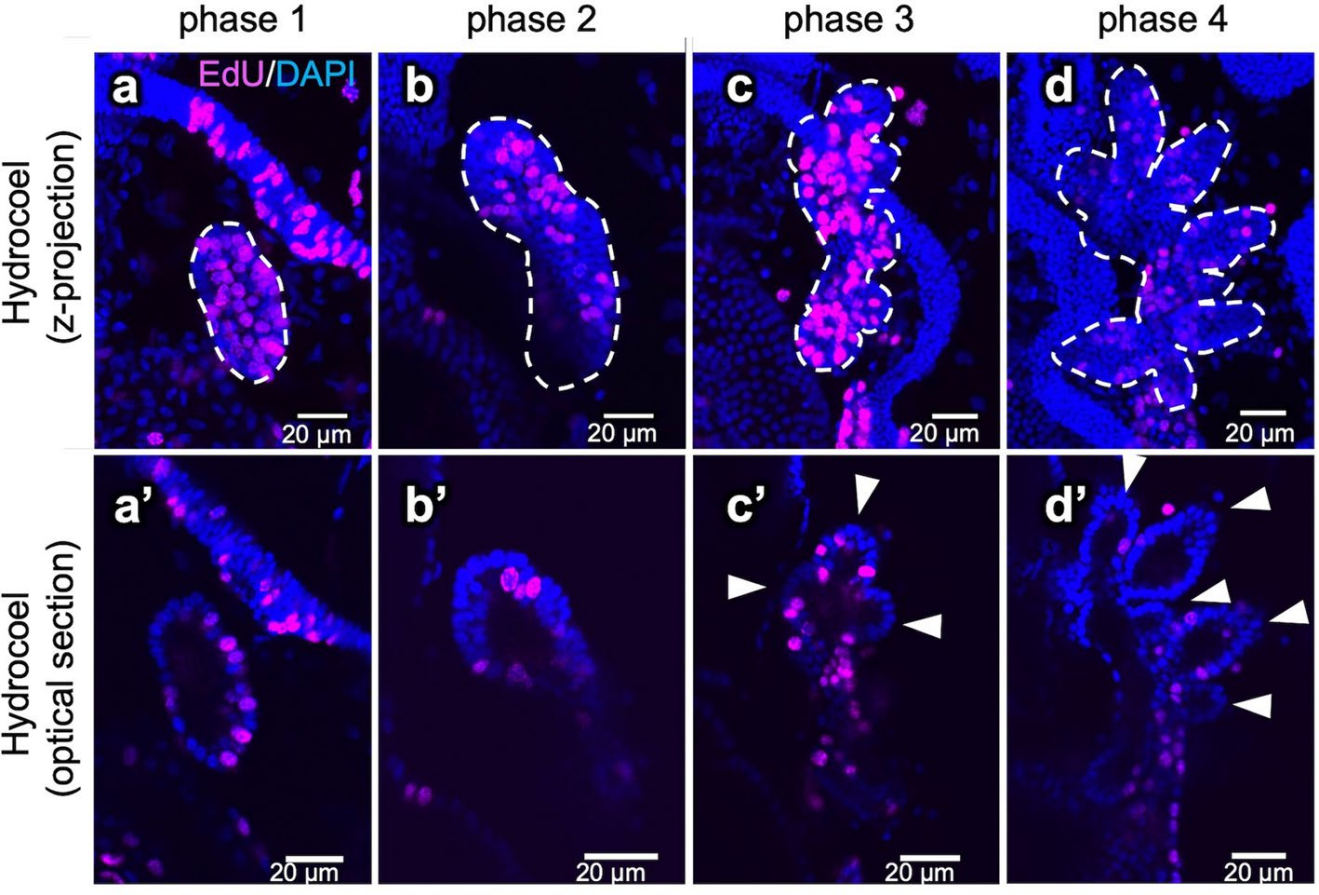
The spatial distribution of cell proliferation in the hydrocoel during the hydrocoel lobe morphogenesis. Cell proliferation in the hydrocoel from phase 1 to phase 4 was detected using EdU after 3 h of EdU treatment (**a–d**). The hydrocoel is indicated by the dotted lines. (**a’–d’**) show the optical sections of (**a**–**c**). The position of the hydrocoel lobe is indicated by arrowheads. The EdU signal was detected equally throughout the whole hydrocoel during phase 1 and phase 4, and specific localization was not observed.

To examine whether cell proliferation contributes to the hydrocoel morphogenesis, we observed the distribution patterns of cell proliferation signals by labeling with 5-ethynyl-2'-deoxyuridine (EdU) during the process. Larvae were incubated in sea water containing 10 µM EdU for 3 h. In all of the observed phases, cell proliferation was detected (Fig. 3).

In phases 1 and 2, the EdU signal was distributed throughout the whole hydrocoel, and observations of both z-stack images and optical sections indicated that the signal was equally localized throughout the hydrocoel (Fig. 3a–b,a’–b’’). The cell proliferation was also observed in phases 3 and 4 (Fig. 3c–d,c’–d’). Both z-stack images and optical sections showed that the EdU signals were not specifically localized at certain regions including lobe tips (Fig. 3c–d,c’–d’, arrowheads).

**Figure 4:**
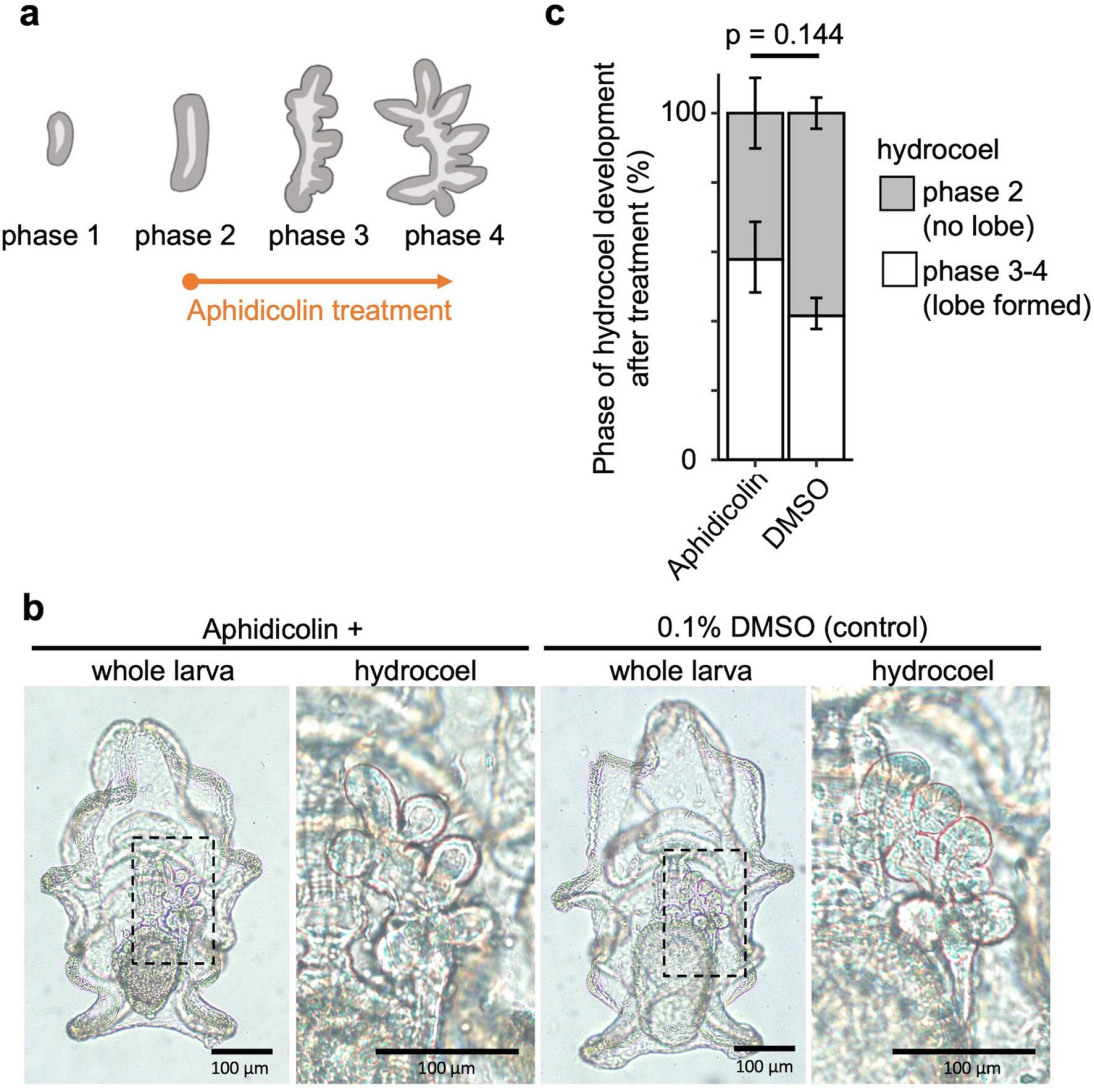
Inhibition of cell proliferation by aphidicolin treatment during the hydrocoel lobe morphogenesis. (**a**) Experimental design for aphidicolin treatment. Auricularia larvae were treated with aphidicolin from phase 2, and the phenotype was observed at 20 h. (**b**) Phenotype caused by aphidicolin treatment. Hydrocoel lobes were formed in both aphidicolin-treated and DMSO-treated (control) larvae. The number or shape of hydrocoel lobes was not significantly affected by aphidicolin treatment compared to DMSO treatment. (**c**) Ratio of phase 3–4 larvae (lobe formed) to phase 2 larvae (lobe not formed) in the treated and control groups at 20 h after the initiation of treatment. There was no significant difference (two-sided Fisher’s exact test, p = 0.144). Graph represents the mean ± s.d. from 3 independent experiments.

To examine whether cell proliferation is required for the hydrocoel-lobe formation, larvae at phase 2 were treated with the cell proliferation inhibitor aphidicolin. Administration of 0.5 µg/mL aphidicolin was shown to completely inhibit the EdU incorporation into hydrocoel tissues, confirming the effect of aphidicolin (Fig. S1). Then auricularia larvae at phase 2, just prior to the hydrocoel-lobe formation, were treated with 0.5 µg/mL aphidicolin (Fig. 4a). At 20 h after the onset of treatment, the hydrocoel lobes were successfully formed, as in untreated animals (Fig. 4b). There was no significant difference in the ratio of larvae in which hydrocoel lobes were formed (phase 3 or 4) to larvae in which hydrocoel lobes were not formed yet (phase 2) between the treated and control groups (two-sided Fisher’s exact test, p = 0.144, Fig. 4c).

### Observations on cell arrangement

**Figure 5:**
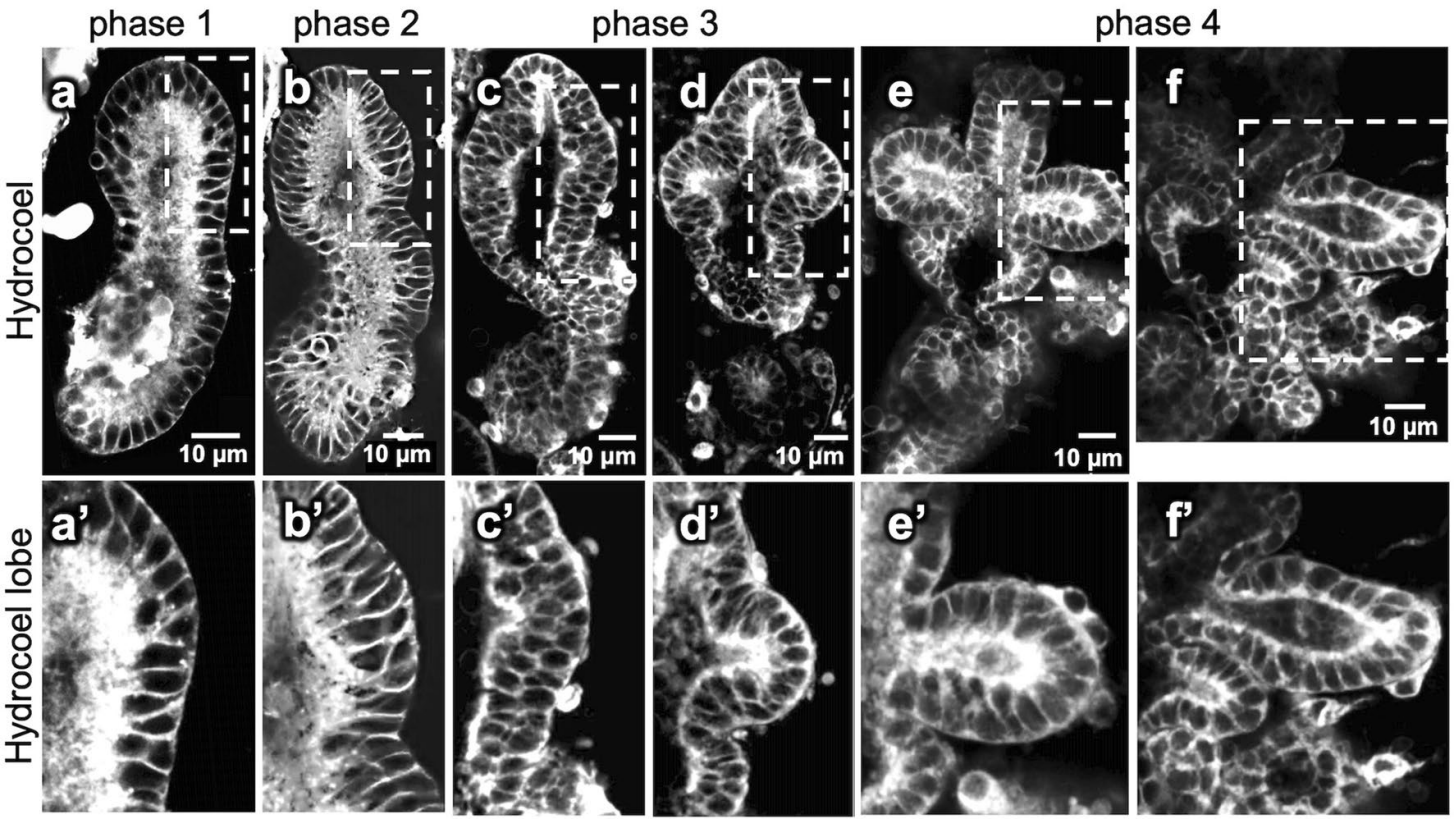
The observed changes in cell shape and cell arrangement during the lobe formation in the hydrocoel. The cell membrane of larvae was stained with BODIPY FL C_5_-ceramide and observed using confocal laser scanning microscopy from phase 1 to phase 4. (**a–f**) Optical sections of the hydrocoel in each phase (ventral view, anterior to the top). The time course and stages are indicated in the figure. (**a’–f’**) show the magnification of the regions indicated in (**a**–**f**), respectively. Hydrocoel epithelium was a single cell layer in phase 1 and seemed to be multilayered in phase 2. In phase 3, the tissue became single layered again at the tip of the lobe (arrowhead in **c**’–**d**’) and then in the whole hydrocoel lobe.

To observe the changes in cell shape and cell arrangement in the hydrocoel epithelium, the cell membrane of live larvae was stained with BODIPY FL C5-ceramide. At phase 1, the hydrocoel was surrounded by a single layer of columnar epithelial cells (Fig. 5a). At phase 2, the hydrocoel epithelium seemed to be multi-layered, which was observed throughout the whole hydrocoel (Fig. 5b). At phase 3, when the lobe formation started, multi-layered epithelial tissue became a simple epithelium again at the tip of the hydrocoel lobes (Fig. 5c–d). The tissue between the lobes was still multi-layered and started to be bent. In phase 4, when the hydrocoel lobes extended, the epithelium between the lobes also became simple epithelium and sharply bent, and consequently, the lobe shape became more constricted (Fig. 5e–f).

**Figure 6:**
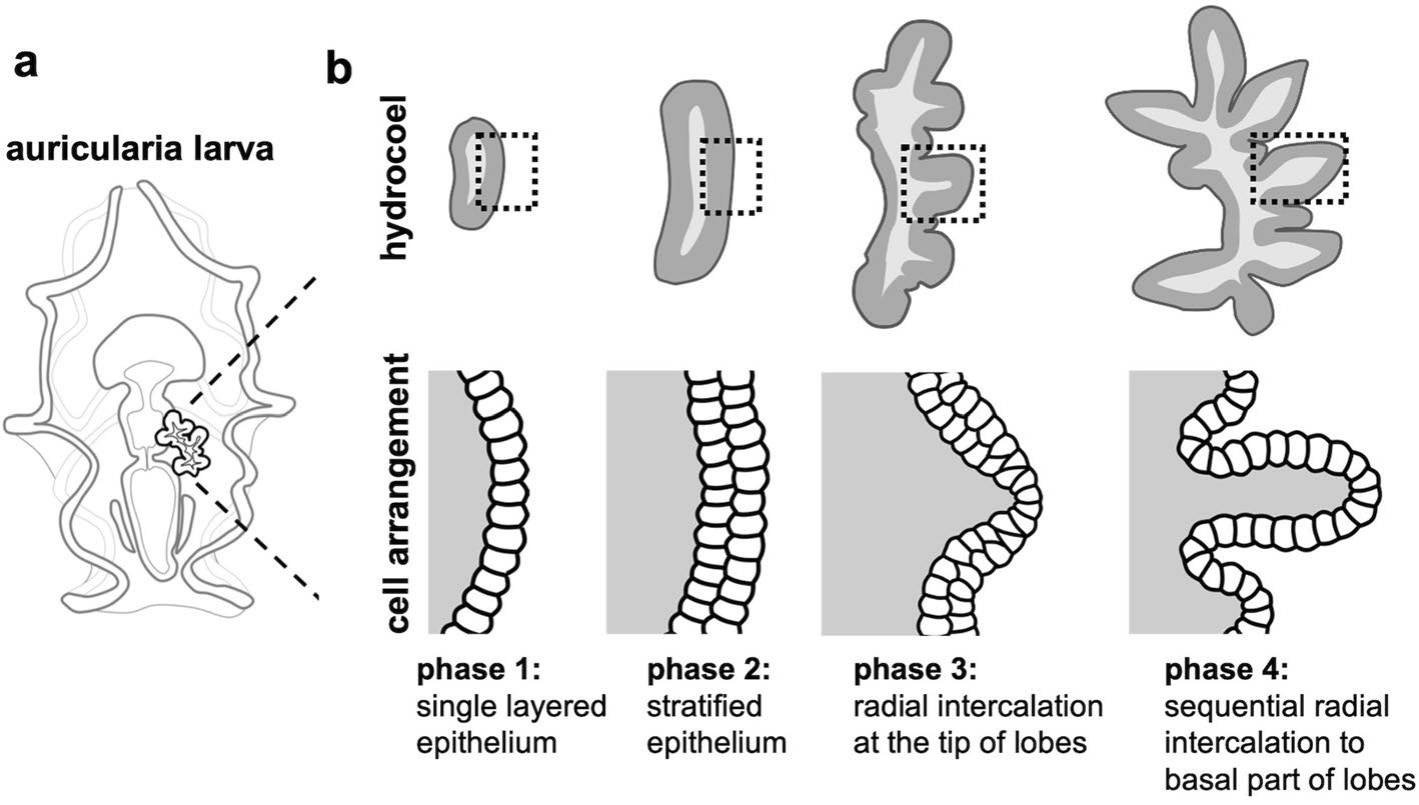
Schematic illustration of the change in cell arrangement during the hydrocoel lobe morphogenesis. (**a**) represents an auricularia larva and (**b**) represents the development of its hydrocoel. The upper panel shows the overall shape of the hydrocoel, and the cell arrangement of the hydrocoel epithelium in dotted areas is shown in the lower panel. The hydrocoel at phase 1 consists of a single cell sheet, and it seemed to be multilayered at phase 2. As the formation of the hydrocoel lobe proceeded, the cell sheet became single layered again sequentially from the tip of the hydrocoel lobe (phase 3) to the proximal part of the lobe (phase 4).

The positions of hydrocoel lobes were the same in all of the observed individuals, and all of the hydrocoel lobes formed synchronously (Fig. 5). Although the lobes for water vascular canals of tentacles (i.e., lobes I, II, III, IV and V) were relatively larger than those for radial canals (i.e., lobes ii, iii, and iv), except for lobe i, the transitions in cell arrangement from multi-layered to monolayered epithelium were observed in all of the lobes regardless of lobe size (Figs. 5, 6).

## Discussion

In general, developmental mechanisms of the formation of branching or budding structures in the epithelium can be classified into three categories, i.e., single cell extension, collective migration and non-migratory branching^19^. Since the hydrocoel lobes are multicellular structures, the mechanism underlying the hydrocoel morphogenesis should be driven by cell migration or non-migratory mechanisms. The non-migratory branching is typically observed during the morphogenesis of vertebrate kidney and mammalian lung^19^. The morphogenesis of ureteric buds in the kidney is driven by localized proliferation at the tip of the branch^19^. In the hydrocoel-lobe formation, cell proliferation was evenly distributed throughout the hydrocoel, and no specific localization of proliferation, such as concentration at hydrocoel-lobe tips, was observed in relation to the lobe formation (Fig. 3b,c). It is still possible that cell proliferation drives the formation of hydrocoel lobes even if no specific localization of cell proliferation was detected. In the case of lung branching in mammals, global proliferation drives the budding process in coordination with cell shape changes and localized muscle cell differentiation^19^. In the hydrocoel-lobe morphogenesis, however, cell proliferation seems to be unnecessary for the hydrocoel-lobe budding, since the inhibition of cell proliferation during phase 2 and phase 4 did not prevent the lobe formation (Fig. 4).

A previous research in a direct-developing sea urchin, *﻿Heliocidaris erythrogramma*, showed that the cell proliferation is not necessary for the formation of the hydrocoel itself^20^. In the hydrocoel formation in *A. japonicus*, however, the thickening and the successive remodeling of epithelial tissue are suggested to be driving forces for the hydrocoel morphogenesis. On the other hand, since cell proliferation is known to be required for the stratification of epithelium^21^, the cell proliferation between phases 1 and 2 is suggested to contribute indirectly to the hydrocoel lobe morphogenesis.

Based on our observations of cell shape and cell arrangement in the hydrocoel, epithelial tissue remodeling is suggested to contribute to budding morphogenesis in the hydrocoel-lobe formation. At phase 1, the hydrocoel epithelium consisted of a simple mono-layered cell sheet, but then the epithelium became thickened, suggesting that it became multi-layered at phase 2 (Fig. 5). The thickened epithelial tissue became thinner again, to be a simple mono-layered arrangement, specifically at the lobe tip (phase 3, Fig. 5). After the epithelium at lobe tip became thinner, same phenomenon was also observed at the proximal part of each lobe (phase 4, Fig. 5). This temporal difference of tissue remodeling among hydrocoel positions suggests that tissue remodeling occurs sequentially from the tip of the hydrocoel lobe to the proximal part.

The thinning of epithelium via tissue remodeling observed during phases 2 to 4 is thought to result in the tissue expansion and the expansion can be a driving force of the hydrocoel-lobe morphogenesis. The structure of thickened hydrocoel epithelium can be stratified epithelium or pseudostratified epithelium since the exact type cannot be determined without observation of fine structures^22^. If the hydrocoel epithelium at phase 2 was stratified, the change in cell arrangement is supposed to be achieved by cell intercalation. Cell intercalation observed in these phases can be categorized as “radial intercalation”, in which cells intercalate in the direction perpendicular to the epithelium and the multiple cell layers are transformed into a single layer^23^. Radial intercalation is thought to lead to tissue spreading^24^, so the tissue expansion caused by sequential radial intercalation might contribute to budding and elongation of hydrocoel lobes. In the branching morphogenesis of the mouse mammary gland, it is known that simple epithelium becomes transiently stratified and is then resolved to simple epithelium again to form the branching structure^25^, and tube elongation is achieved by radial intercalation^24^. Hydrocoel lobe morphogenesis resembles these processes. It is also known that tissue remodeling from pseudostratified epithelium to columnar epithelium leads to tissue expansion in pseudostratified epithelium^22^. This phenomenon also resembles to the changes from phase 2 to phase 4 in the hydrocoel tissue.

To further understand the detailed mechanisms of hydrocoel-lobe formation, observations on the fine structures of hydrocoel epithelia and functional assays to examine whether the remodeling of epithelium is the driving force of morphogenesis should be required. The tissue was curved sharply at the tips of hydrocoel lobes and between the hydrocoel lobes as the hydrocoel lobes developed, although it is not clear whether these changes in epithelial tissue constitute an active process to form the hydrocoel lobes or a passive result of morphogenetic processes such as tissue expansion.

It is known that tissue expansion via radial intercalation is driven by cell chemotaxis in amphibian epiboly^26^. Therefore, in the focal sea cucumber, sequential tissue remodeling during the hydrocoel-lobe formation could also be driven by chemotaxis. If so, the diffusion pattern of attractants and/or the localization of their source might determine the number and the position of hydrocoel lobes. Alternatively, specific localization of gene expression might regulate the responsiveness of epithelial cells to the attractants, leading to the sequential tissue remodeling. Future studies on the molecular mechanisms involved in hydrocoel-lobe morphogenesis will help us to understand the developmental mechanisms underlying the pentaradial body plans in echinoderms.

## Methods

### Animals

Mature adults of *A. japonicus* were collected between April and June of 2019–2021 from the sandy bottom of the sea at a depth of 3–10 m around the Misaki Marine Biological Station, Kanagawa or at Onahama Port, Fukushima (36° 94′ N, 140° 90′ E) by SCUBA diving. All the collected individuals were morphologically diagnosed as the black type of the focal species, which was recently redescribed as *A. armatus*^27^, although it is still treated as *A. japonicus* in many recent studies*.* Collected individuals were kept in the laboratory tank at 17 °C to maintain the sexually mature conditions. Gonads of collected individuals were dissected from inside the body wall to discriminate sex and maturity. Mature ovary is vivid orange and mature testis is white. Egg spawning was induced by the injection of 500 µl of 10 µM cubifrin (Kyusyu TLO Company, Fukuoka, Japan), a peptide known to induce oocyte maturation and spawning in *A. japonicus*^28^. Sperms were obtained by dissecting mature testes in sea water.

Embryo culture was conducted based on Kikuchi et al.^29^. Briefly, embryos were kept in 10 L of filtered sea water in a plastic container with circulation at 20 °C and fed *Chaetoceros calcitrants* diatoms daily. The density of individuals was kept at a embryo/mL, and sea water was changed every two days. Embryos become bilateral auricularia larva at 2 dpf. The hydrocoel emerges at the left side of the digestive tract at approximately 6 dpf, and the hydrocoel lobes can be observed at approximately 10 dpf^11^. Individuals during this period were used for observations. After the formation of hydrocoel lobes, larvae metamorphose into doliolaria and pentactula juveniles and settle down on the container bottom.

### Morphological observation of the hydrocoel

For observations of the overall shape of hydrocoel, larvae were fixed using Fix1 (4% PFA/0.1 M MOPS, pH 7.0/0.5 M NaCl)^29^). Specimens were stained with DAPI (Nakalai Tesque, Kyoto, Japan) to observe the overall morphology of the hydrocoel. For observations of cell arrangements and cell shapes, *N*-(4,4-Difluoro-5,7-Dimethyl-4-Bora-3a,4a-Diaza-s-Indacene-3-Pentanoyl) sphingosine (BODIPY FL C_5_-Ceramide) (Life Technologies, Carlsbad, CA, USA) was used. Larvae were kept alive in BODIPY FL C_5_-ceramide (100 μM in sea water) for 6 h at 20 °C. Based on a previous study^30^, specimens were embedded on glass bottom dishes using 1% low-temperature melting agarose. The specimens were then observed using a confocal laser scanning microscope (FV3000, Olympus, Tokyo, Japan) and an optical microscope (TE300 Nikon, Tokyo, Japan) equipped with a camera (DP27, Olympus, Tokyo, Japan).

### Detection and inhibition of cell proliferation

To observe the localization of cell proliferation, the intake of the thymidine analogue 5-ethynyl-2'-deoxyuridine (EdU) was detected using a Click-iT™ EdU Cell Proliferation Kit for Imaging (Life Technologies, Eugene, OR, USA). EdU labeling was conducted based on Sharma & Ettensohn^31^ with some modifications. EdU was diluted to 10 μM in filtered sea water according to the manufacturer’s instructions. Auricularia larvae at each phase were incubated in 10 mL of EdU sea water for 3 h at 20 °C. Larvae were fixed with Fix1 soon after the treatment. Detection of EdU was conducted based on the manufacturer’s instructions, and cell nuclei were stained with DAPI. Then, the stained specimens were observed using a confocal laser scanning microscope.

Cell proliferation was inhibited using the DNA polymerase I inhibitor aphidicolin (Adipogen, Liestal, Switzerland). This reagent is known to inhibit cell proliferation in a sea urchin, *Lytechinus pictus*^32^, as in many other eukaryotes. The treatment method was based on Smith et al.^21^. Aphidicolin was dissolved at a concentration of 10 mg/Ll in dimethyl sulfoxide (DMSO) and diluted to 0.5 µl/mL in filtered sea water. Auricularia larvae before lobe formation were incubated in aphidicolin sea water for 20 h at 20 °C. Control experiments were conducted by incubating under the same conditions using 0.5 µl/mL DMSO in filtered sea water. For the evaluation of the effect of aphidicolin on the hydrocoel-lobe formation, phase 2 larvae were randomly divided into six groups. Three of the groups were kept in aphidicolin sea water, and the other 3 groups were kept in DMSO sea water (control). Larvae were fixed after 20 h of treatment, and the number of larvae that formed the hydrocoel lobes (phase 3 and phase 4) was counted (10–18 larvae in each group). Two-sided Fisher’s exact test was carried out with R 4.0.3.

## Supplementary Information

Supplementary Information.
