# Supplementary material for: Hydrocoel morphogenesis forming the pentaradial body plan in a sea cucumber, Apostichopus japonicus
Source: Sci Rep. 2022 Apr 11;12:6025. doi: 10.1038/s41598-022-09691-y (PMC9001670; doi:10.1038/s41598-022-09691-y)
Supplement: Supplementary file 1 — Supplementary Information. [file 41598_2022_9691_MOESM1_ESM.pdf]

Supplementary Figure

Hydrocoel morphogenesis forming the pentaradial body plan in a sea cucumber, Apostichopus japonicus

Sumio Udagawa, Takafumi Ikeda, Kohei Oguchi, Hisanori Kohtsuka and Toru Miura

Supplementary figure 1

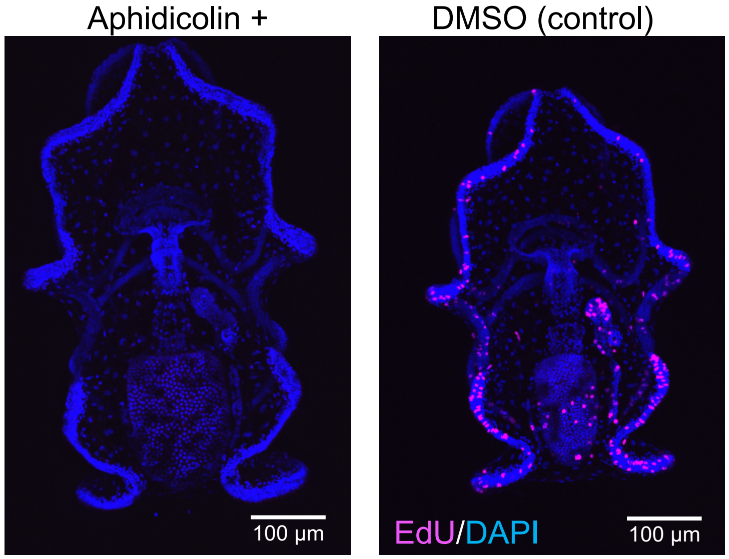

Supplementary Figure 1 EdU detection in aphidicolin-treated specimens.
Cell proliferation was detected using EdU after a 3-hour treatment with aphidicolin. In larvae incubated with EdU and 0.5 % DMSO (control), EdU signal was detected in the whole body. On the other hand, EdU signal was not detected in larvae incubated with EdU and aphidicolin.
